# Semi-automatic spike sorting with high-count channel probes

**DOI:** 10.1186/1471-2202-14-S1-P160

**Published:** 2013-07-08

**Authors:** Cyrille Rossant, Kenneth D Harris

**Affiliations:** 1 Institute of Neurology, University College London, UK

## 

Automatically extracting spiking information from extracellular recordings is a fundamental but still unresolved issue in experimental neuroscience, despite decades of efforts [1]. A fully automatic spike sorting algorithm appears to be out of reach today, mainly because of the large diversity in experimental settings and protocols. Besides, in-vivo recordings are typically highly noisy, making extremely difficult the separation of neuron sources in the spiking activity. Additionally, the development of new silicon probes with a very high number of channels raises new computational problems that require novel approaches [2].

Most existing methods are based on semi-automatic algorithms, with a first, automatic step and a second, manual step requiring the neurophysiologist's interaction. The manual step is typically required because no algorithm yet has the required expertise to check the quality of a spike sorting result. The experimenter is then given a chance to check, validate, and refine the original result. This step can be particularly long and may prevent the neurophysiologist from focusing on the scientific questions of interest underlying his experiments.

We are developing a set of tools that aim at making spike sorting sessions as efficient as possible in terms of computer time, human time, and sorting quality. We are currently focusing on improving the manual step by developing a new ergonomic graphical user interface. This interface guides the user through the automatic algorithm's output and asks him to make decisions about ambiguous clusters of spikes. Similar clusters that are likely to stem from the same neuron according to a probability metric are automatically selected. The software also chooses automatically the best feature projection that maximizes the distance between the clusters. The user can then decide whether these clusters should be effectively merged. Several interactive views on the data are available to help the user make the best decisions. Together, these steps allow to improve the resulting spike trains quality.

We have chosen to develop this suite of tools in Python, an increasingly used language in the scientific community [3]. Performance is achieved through thin bindings around highly optimized low-level libraries such as Numpy (vectorized computations), HDF5 (efficient random-access input/output), Qt (graphical user interface) and OpenGL (open and portable hardware-accelerated visualization library).

Our approach should make possible the analysis of coordinated spiking activity across hundreds of neurons in in-vivo recordings, a crucial step in understanding the neurophysiological bases of behavior.
